# Co-designing postpartum contraceptive services with and for immigrant women in Sweden: lessons learned from the IMPROVE-it project

**DOI:** 10.1186/s12913-024-11709-2

**Published:** 2024-10-31

**Authors:** Sibylle Herzig van Wees, Helena Kilander, Khadija Salah, Sahra Saidarab, Anna Wängborg, Elin C. Larsson

**Affiliations:** 1 Department of Global Public Health, Karolinska Institutet, Stockholm, Sweden; 2 Department of Women’s and Children’s Health, Karolinska Institutet, Karolinska University Hospital, Stockholm, Sweden; 3 Jönköping Academy for Improvement of Health and Welfare, School of Health and Welfare, Jönköping University, Jönköping, Sweden; 4 Department of Women’s and Children’s Health, Uppsala University, Uppsala, Sweden

**Keywords:** Co-design, immigrant populations, midwifery, contraception, postpartum contraception, Sweden

## Abstract

**Background and aim:**

Immigrant women in many high-income countries including Sweden, report unmet need of sexual and reproductive health and rights, and face worse pregnancy outcomes and higher risk of unintended pregnancies. Postpartum contraceptive services are often inadequate to meet their needs. Co-design has shown to reduce health inequities, yet little is known about using this method for postpartum contraceptive service development and even less in collaborating with immigrant populations. The aim of this paper is to describe the co-design process and the strategies that were developed to help develop tailored and acceptable postpartum contraceptive services for immigrant women in Sweden.

**Methods:**

The paper describes a co-design process that took place during 2022–2023, including the cyclical digital consultations with Arabic and Somali speaking immigrant women, midwives and researchers, as well as the outputs from the process. The theoretical framework for the co-design process was the ‘Double Diamond’ Design Process Model. Data analysis included qualitative content analysis.

**Results:**

The co-design process led to the joint development of intervention materials and strategies to improve postpartum contraceptive services. Specifically, the process revealed ideas on how to improve contraceptive counseling within three pre-identified areas of change: improve physical access to contraceptive services; improved communication strategies using visual aids and information charts; and empowerment strategies that focus on reflective practice without assumptions about what a group of women might expect. We found that participants contributed actively to the process with ideas and suggestions, and that the co-design process facilitated positive reflections on ongoing counseling practices.

**Conclusion:**

The co-design process resulted in the successful and participative development of innovative tools and activities to improve contraceptive counseling services. This approach is original because it involves both immigrant women, often left behind, and midwives delivering the services. Whilst this interplay allowed for careful refinement of services and tools by using an iterative process, it also facilitated reflective midwifery practice.

**Supplementary Information:**

The online version contains supplementary material available at 10.1186/s12913-024-11709-2.

## Background

Universal access to sexual and reproductive health and rights (SRHR) including contraception prevents unintended pregnancies, abortions, enables birth spacing and contributes to essential women’s health and wellbeing [1]. Access to postpartum contraception is particularly important because birth spacing of more than 12 months can reduce the risk of complications such as preterm birth, low birthweight, and neonatal death [1–4]. Evidence further suggests that women in general appreciate postpartum contraceptive counseling and that healthcare workers consider it feasible and effective [5, 6]. Yet, immigrant women from low- and middle-income countries living in high-income countries report lower contraceptive use compared to native-born women [7, 8] and are more likely to use less effective contraceptive methods, such as natural family planning or barrier methods [7–11]. They also describe more negative experiences of postpartum contraceptive counseling [12–14], language barriers, limited partner involvement or support [15] and challenges in accessing appropriate information about contraception [16, 17]. However, a strong focus on improving contraceptive use has been experienced as pressuring immigrant communities [18–20]. Therefore, this study focuses on addressing unmet need, by involving immigrant communities in the development and design of tailored interventions.

Co-design in global health involves a participatory approach to the development of health interventions and brings together experts such as researchers and healthcare professionals and clients or end-users to design tailored solutions to context specific needs [21]. Co-design processes harness the collective creativity of all participants involved to create a service that suits everyone’s needs and local ownership, considering the diverse perspectives from diverse groups. Co-designed solutions are more likely to be accepted and used by the end-users [22]. Research on the use of co-design in healthcare settings has been growing over the past decade. It has been applied to develop digital health interventions, and interventions to improve services for patients with chronic diseases [23]. The co-design process in this study was inspired by the Design’s Council Double Diamond [24]. The two diamonds represent a process of exploring an issue innovatively and then taking focused action [24]. It involves four principles: The first diamond includes the principles of Discovery and Definition. This helps to understand people rather than merely assuming what the issue is. The insights gathered from the discovery phase help define the challenges innovatively. The second diamond involves the process of Development and Delivery. In the development phase, people who are part of the process are encouraged to provide their responses to defined problems. In the delivery phase, different solutions are tested at a small scale, rejecting ideas that do not work and improving solutions that will [24]. A co-design process is not linear but rather iterative, meaning that ideas and solutions may continuously change and require adaptation.

Evidence on effectiveness of co-design is limited, yet evaluative reports suggest that the methods have been successful in improving health care services and outcomes [21]. Less research is available on co-design involving immigrant populations and of interventions to improve contraceptive services.

In this paper, we describe the co-design process inspired by the Double Diamond model to develop tailored interventions to improve postpartum contraceptive services targeting immigrant women and midwives in Sweden. The aim of this paper is to describe the co-design process and the strategies that were developed to help develop tailored and acceptable postpartum contraceptive services for immigrant populations in Sweden.

## Methods

### Context

In Sweden, approximately 30% of women who give birth are foreign born [25]. Whilst the foreign-born population in Sweden is heterogenous, the largest groups are immigrants from Syria, Iraq, Iran and Somalia [26]. All women who give birth in Sweden are offered postpartum visits to a midwife at maternal health clinics (MHCs) within 16 weeks postpartum, which also includes contraceptive counseling [27]. In Sweden, nurse-midwives provide most contraceptive services [28, 29]. Contraceptive counseling is available to all ages and free at point of service for Swedish citizens and those with a residence permit, including asylum seekers. Several hormonal contraceptives, such as the levonorgestrel-containing intrauterine system and subdermal implants, are subsidised up to the age of 25 years [29].

There are some regions in Sweden where digital contraceptive counseling is available. However, this was beyond the scope of this project because postpartum contraceptive counseling is offered in person.

### Contextualize the co-design: description of study area and project (IMPROVE-it)

This project is part of a large-scale intervention and trial (IMPROVE-it) that uses a Quality Improvement Collaborative (QIC) including co-design and tailored interventions to improve the experience of postpartum contraceptive counseling and subsequently facilitates women’s choice of postpartum contraceptive methods [30].The IMPROVE-it study builds on a pilot study which shows that a QIC could increase immigrant women’s uptake of postpartum contraception [31]. This paper focuses on the co-design process of this complex intervention. The co-design process was an integral part embedded in and continuously provided input to the QIC.

### Conceptual framework to guide the co-design process

The theoretical framework for IMPROVE-it and thus the co-design process was based on the hypothesis that by focusing on three evidenced-based areas of change, contraceptive services will improve and support immigrant women in choosing an effective postpartum contraceptive method that suits their individual needs [31]. The three evidenced-based areas of change involved: (1) Developing tools to share information about contraceptive methods’ effectiveness; (2) Developing respectful approaches to provide contraceptive counseling; (3) Improving access to all contraceptive methods [31]. We used these areas to guide the development of the improvement activities during the co-design process.

### Objectives of the co-design process

In line with the Double Diamond model, the overall co-design process sought to:

1. Create a shared understanding of unique attributes of postpartum contraceptive counseling from the perspective of immigrant women and midwives (*Discover*).
2. Create a shared understanding of the barriers and enablers to effective postpartum contraceptive counseling from the perspective of immigrant women and midwives *(Define*).
3. Identify and develop strategies to improve postpartum contraceptive counseling (*Develop*).
4. Develop feasible and acceptable strategies to improve postpartum contraceptive services *(Deliver).*

### Co-design procedures

The co-design process took place between April 2022 and November 2023. It included 13 focus group discussions (FGD) and four co-design workshops with Arabic-speaking and Somali-speaking women with lived experiences of contraceptive services from the three regions in Sweden included in IMPROVE-it. In addition, the co-design process included four digitally run learning sessions (LS) with midwives from 13 maternal health clinics (MHCs) from three out of the 21 regions in Sweden. The process included action periods between the LSs where participating midwives applied learnings and tested co-designed tools during antenatal and postpartum visits at the MHCs.

### Participant recruitment

Somali and Arabic-speaking women were invited to participate in FGD and digital co-design workshops with researchers to share their experiences of contraceptive services, including postpartum contraceptive counseling. The details and results of the FGDs are described elsewhere (studies currently under review). However, the FGDs were important for knowledge generation to help inform the co-design process. A group of eight Arabic-speaking and twelve Somali-speaking women were recruited to participate in a series of two co-design workshops for each language (4 co-design workshops in total), each described in detail in the Results section. Some Arabic-speaking participants participated in both workshops, whereas different participants were recruited for the co-design workshops with Somali-speaking women. Co-design workshops were led by a Somali and Arabic speaking researcher. We led two separate series of co-design workshops for pragmatic reasons due to language but also to explore potential socio-cultural differences between the groups.

46–55 midwives from 13 MHCs were participated in the four learning seminars (LS) as part of the wider IMPROVE-it project (see Table 1).

**Table 1:** Overview participants and data collection co-design process in IMPROVE-it

Source	Participants	Data collected	Date
Focus group discussions	• Arabic speaking women (*n* = 24) • Somali speaking women (*n* = 60) • Researchers (*n* = 4)	Transcripts	April 2022-November 2022
Learning sessions 1–4	• Midwives (*n* = 46–55) • Researchers (*n* = 3) • Research assistant/doctoral student (*n* = 2)	Audio recordings, material shared, notes	December 2022, February, May & September 2023
Workshop 1–2	• Arabic speaking women (*n* = 3) • Researchers (*n* = 3) • Arabic speaking research assistant (*n* = 1)	Notes, audio recordings	January & May 2023
Workshop 3–4	• Somali speaking women (*n* = 6) • Somali speaking midwife outside the project (*n* = 1) • Researchers (*n* = 3)	Notes, audio recordings	January & May 2023

### Data analysis

**Fig. 1:**
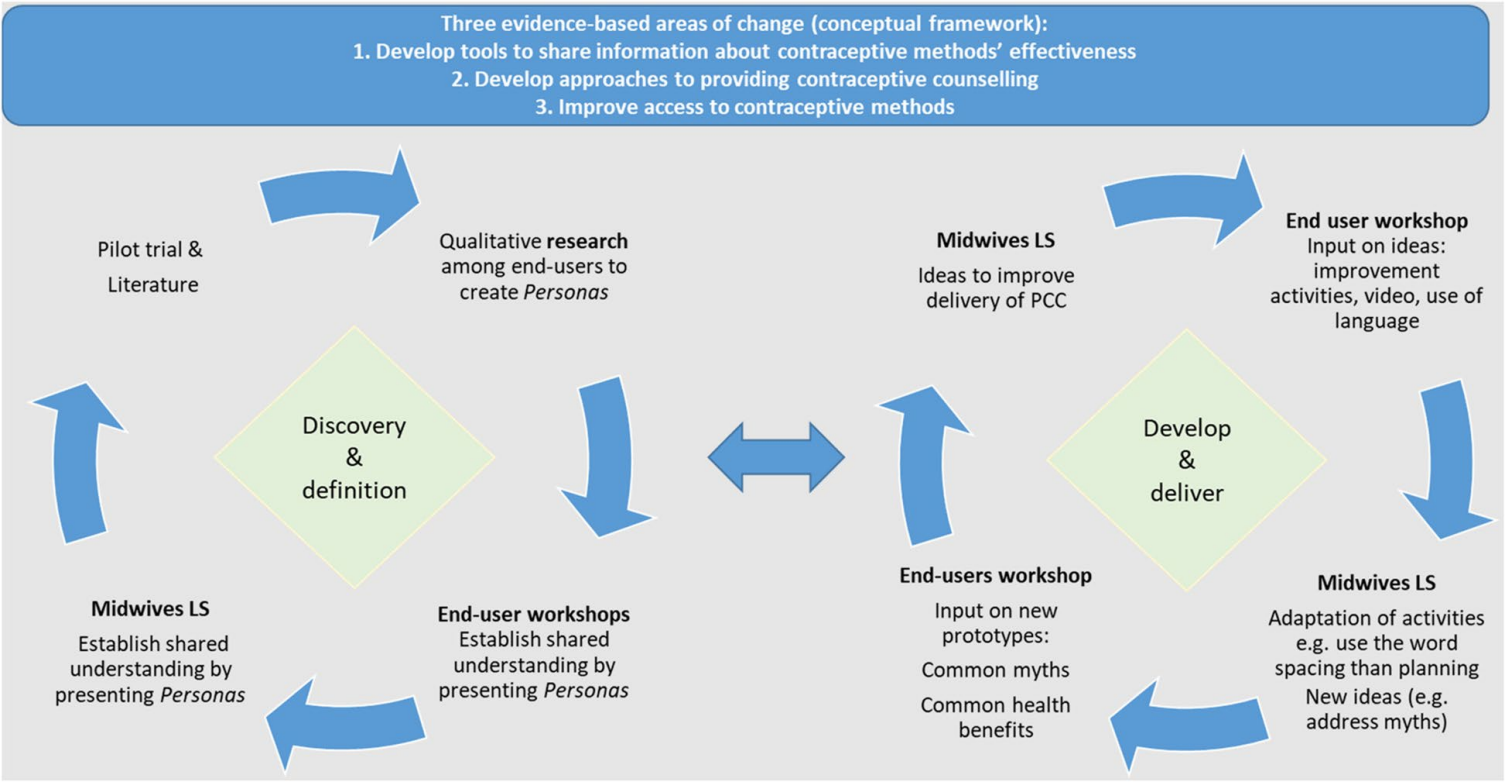
The co-design process inspired by the Double Diamond

We applied content analysis to analyze data from the co-design process [32]. Transcripts, workshops notes, and PowerPoint presentations from LS and workshops were reviewed by the authors. SHvW conducted a deductive content analysis of key materials pertaining to the specific research questions applying the Double Diamond model and the three evidence-based areas of change. As per Elo and Kyngnäs [32] the content analysis included familiarization and organization of data. Data coding and the creation of categories and themes was possible when data was sufficiently rich as per Tables 2 and 4. The co-design team reviewed the analysis, and the key findings were agreed upon through a cyclical process of several discussions. The results from the analysis are described in Fig. 1, and Tables 2, 3 and 4. We initially separated analysis for Somali and Arabic speaking groups to allow for exploration of socio-cultural specificities. While we found some differences between the groups as described in Table 2, the co-designed strategies were welcomed by both groups and we therefore did not differentiate between them.

**Table 2:** Discovery and definition of immigrant women’s experiences of postpartum contraceptive services – generated through discussions about Personas

Somali speaking	Arabic speaking
Feeling forced or talked into contraception; feeling of being belittled.	Generalized fear of side effects from contraceptives, particularly hormonal contraceptives.
Difficult to focus on contraceptives during pregnancy. Contraception discussion appeared far too early after delivery. The focus should be on the current pregnancy and baby.	Limited knowledge about contraceptives as well as about sex education in general.
Concerns over not being able to get an appointment to take contraceptive out if one changes their mind. Experience negative side-effects	Culture and assumptions about culture should not be at the center of the counseling. Experience negative side-effects

**Table 3:** Discovery and definition of midwives’ perspectives of challenges regarding postpartum contraceptive counseling for immigrant women

Midwives’ challenges to contraceptive counseling for immigrant women	
• Limited time because encounter is more complex • Challenges working with an interpreter (sometimes poor quality) • Common queries by women are related to how to navigate the health system and other questions not related to contraceptive counseling • Contraceptive counseling is not a priority among many immigrant women • Difficult to communicate when basic knowledge of female reproductive health is not there • Religion and culture influence reproductive decision-making

**Table 4:** Co-designed strategies to support the development of postpartum contraceptive services in the IMPROVE it project

Evidence-based areas of change [30]	Definition	Implementors	Target	Key strategies/actions
Improving access to all contraceptive methods	This refers to activities that make best use of limited time of the CCs	Midwives	Women and partners	Share videos on contraception to prepare women (and partners)
		Midwives	Women	Longer appointments
Developing tools to share information about effectiveness of contraceptive methods	This refers to a series of tools or aids that were developed to help improve the communication between midwives, women and partners and to improve the CC experience.	Midwives	Women	Use of models of contraceptive methods
		Midwives	Women	Effectiveness chart
		Midwives	(Women)	Myth chart
		Midwives	(Women)	Health benefits chart
		Midwives	Women and partners	Video on contraception
		Midwives	Midwives	Webpage with all materials summarizing tools and evidence-based materials
Developing respectful approaches to provide contraceptive counseling	This refers to concrete strategies to improve language with the aim to build trust and achieve an empowering CC experience.	Midwives	Midwives	Supportive language, do not try to convince, consider own ideas and experience of women
		Midwives	Midwives	Reflective of language and bias
		Midwives	Midwives	Consent for CC and timing of CC
		Midwives	Midwives, women and partner	Invite partner to CC

### Ethics

The study was approved by the Regional Ethical Review Committee in Sweden. The reference number is 2021-05962-01. We followed the principles of the Helsinki Declaration. All participants gave their written informed consent. Through the consent process, participants agreed to recording of the FGDs or LSs. Participants were renumerated with the equivalent of an hourly paid research assistant salary.

## Results

### Description of the co-design process

The co-design process consisted of an iterative process of the two cycles as per the Double Diamond Model [24]. The workshops with immigrant women and the LS with midwives happened in sequences. The co-design process is visually presented in Fig. 1.

### Discovery & definition phase

In an initial discovery and definition phase, we drew on three elements to help define the research question: previous research and literature, consultation workshops with midwives delivering postpartum contraceptive counseling to immigrant women, and qualitative research with Somali and Arabic speaking women [31, 33].

### Pilot trial and literature

Members of the research team were involved in a pilot trial in 2022, which described the importance of developing tailored contraceptive services for immigrant women in Södertälje, Sweden [31]. The small-scale quality improvement collaborative supported by register and end user feedback, helped midwives develop postpartum contraceptive services. The study reports positive results when aiming to improve choice of more effective contraceptive methods among immigrant women but did not evaluate women’s satisfaction with the counseling and actual use [31]. However, the co-design process in that project was not well developed. Due to well documented power imbalances between health care professional and immigrant end users [31, 34], we decided to have separate and homogenous workshops and learning seminars in the co-design process used in this study.

While research on migrant’s perspectives on contraceptives exists, limited research is participatory in nature. Moreover, a review of immigrant’s involvement in participatory research has highlighted that immigrant communities have collaborated on several health-related studies but have rarely been involved in all stages of the research process, especially at the level of implementation [35, 36].

### Qualitative research

The results from the two separate qualitative studies with Arabic and Somali speaking immigrant women respectively highlighted that immigrant women experience barriers to contraceptive services (forthcoming publications). They further describe common barriers to accessing contraceptive services such as lack of trust in the individual meeting and logistical obstacles. This was exemplified with prejudice and preconceived notions among midwives they met, for example the assumption that Somali women do not want to use contraceptives as they give birth every year. Organizational barriers included difficulties in obtaining an appointment when experiencing negative side-effects of the chosen method or wanting to remove methods used. The literature and the qualitative research data was used to help define and discover the problem.

### Personas

Moreover, we used the qualitative data to develop *Personas*, which aim to illustrate typical situations of immigrant women’s experience of contraceptive services. A *Persona* is a commonly used co-design tool and allows participants to share their views by creating a fictional person [34]. *Personas* can foster creative thinking among professionals and are used to address needs and motivations of a specific group in a community [37]. In this study, we developed two *Personas* of a Somali and an Arabic-speaking women each based on the qualitative research. They summarize common stories and patterns we found in the qualitative data (see appendix/supplement).

### Workshops with immigrant women

We conducted separate workshops with Somali and Arabic-speaking immigrants. In the workshops we presented the *Personas*, thereafter, the participants had the opportunity to comment on their interpretation. It was also an opportunity to establish common notions of experiences of postpartum contraceptive counseling among immigrant women. Their feedback and reflections on the *Personas* are summarized in Table 2:

The *Personas* were revised based on the feedback and presented to the midwives in the midwives learning seminars (LS).

### Midwives learning seminars

Multiple LS with midwives were conducted to discover and define the problem (first diamond). In the first seminar we asked midwives to share their experiences of postpartum contraceptive counseling (CC) with immigrant women, hence discovery and definition of the problem (first diamond). In a LS we identified the following experiences (see Table 3):

In the second LS the midwives were presented with *Personas*, described above. Midwives then got the opportunity to comment and share reactions. Overall, midwives felt that the *Personas* were an accurate reflection of their clients and contraceptive counseling experiences. Although some depictions of midwives were negative, midwives did not take offence and instead exhibited self-reflection at an individual level and a professional level. Midwives would agree with some of the negative depictions of them and their colleagues and rather than being defensive, they saw it as an opportunity to challenge their notions and practices.

### Development & delivery

The *Definition* and *Discovery* phase allowed to create the foundational knowledge to help develop the strategies to improve postpartum contraceptive counseling. We used the evidence-based areas of change and knowledge from the first co-design phase to develop strategies to improve the postpartum contraceptive services for immigrant women and midwives. In a cyclical process, the research team presented core questions and practical solutions to the Somali and Arabic-speaking immigrant women and midwives. Their input was then used to revise content and materials, until all participants agreed on final strategies and solutions to improve postpartum contraceptive services. The outputs from the co-design process include strategies to develop postpartum contraceptive services for an improved experience for both midwives and immigrant women as summarized in Table 4.

### Participant engagement

We reached a good level of participant engagement of both midwives and immigrant women. This may be because all members were given incentives to participate. Engagement was active in that participants included multiple ideas and thoughts. The online format worked well even though online engagement can at times lead to lower engagement. It may also be that engagement was high because we separated groups into midwives and two different language groups. This separation arguably created a safe space. However, diverse levels of education may have led to some participants being more engaged or confident than others. Moreover, the arrangement of payment of incentives was complicated for some participants due to bureaucratic challenges and may have led to a disincentive to continue participation in further discussions.

## Discussion

In this paper we described the process of how we used co-design approaches to improve three areas within contraceptive services (1), improving access to all contraceptive methods; (2) developing tools to share information about contraceptive methods’ effectiveness; and (3) developing respectful approaches to provide contraceptive counseling. The co-design process led to the development of multiple strategies within these evidence-based areas of improvement with the aim to improve postpartum contraceptive services for both immigrant women and midwives. Within the first area of improvement i.e. to increase access to contraceptive methods, the co-design process led to the idea of providing information prior to the counseling session to allow taking time to review information and come to a session prepared. This preference was previously described in the literature where clients highlighted the value of receiving information prior to consultation in the USA [38]. Moreover, all participants highlighted the importance of longer appointments for immigrant women to allow to address all concerns and questions while also working with an interpreter. This echoes literature from the USA where the effect of longer and tailored contraceptive counseling sessions have been proven to increase contraceptive use [39].

Within the second evidence-based areas of change, we developed tools to share information about contraceptive methods’ effectiveness including use of visual aids and charts as described in Table 4 in detail. This is in line with literature that highlights women’s preference for information in different forms, particularly visual form and seeing examples of contraceptive methods [12, 40]. The co-design process also highlighted how midwives appreciated more support tools during their consultation.

Within the third area of evidence-based area of improvement, i.e., by focusing on developing respectful approaches to provide contraceptive counselling, we identified multiple strategies from the co-design process. Participants highlighted the importance of use of language. As an example, spacing was a more acceptable term instead of using words such as prevent a pregnancy. This is also in line with previous research [41]. Furthermore, midwives should not try to convince clients. This echoes literature from the USA where ethnically diverse clients felt that healthcare providers were coercing them into the use of postpartum contraceptives [42]. This could be further supported by a strategy described which includes the importance of asking for consent to conduct the counseling, rather than merely assuming that women want postpartum contraceptive counseling in the first place. Moreover, both midwives and women felt that it is important to be conscious of own bias, this has been described elsewhere where it was found that midwives shall remain curious, positive, and open about diverse cultural values related to reproductive health and choices, rather than labelling. Linked to this was that some women felt that the partner should also be involved in family planning decisions. This is in line with similar findings from Sweden, where immigrant men have shown interest to be involved in contraceptive counseling and decision-making [43].

This co-design study is novel in multiple ways. First, it included immigrant populations as well as midwives, which adds to existing and limited literature of engaging immigrant populations in research in general and in co-design in particular research [41]. We used *Personas* to structure discovery and definition of the problem from the perspectives of multiple actors. We successfully used online engagement to facilitate inclusion of a wide range of actors.

Moreover, we concluded that the co-design process including *Personas* facilitates a conversation that is otherwise difficult. Midwives did not react in a negative or hostile way to what could be perceived as accusations. This is in line with research from Sweden where case studies about individuals were developed which successfully facilitated conversations about racism in the healthcare setting [44].

Lastly, our study contributes with new findings how to engage seldom heard groups in all steps of the research process to improve contraceptive services. Many studies have been carried out to improve SRHR services, but immigrant groups’ are rarely involved in the research process [44], especially in co-design and the later implementation phases [35, 36, 45]. Establishing partnership with neglected groups and balancing power issues are central in co-design and co-production of services [35, 45, 46]. Women participated actively in the co-design process and there were few dropouts during the process, which could be interpreted as successful regarding power dynamics i.e. participants felt included. However, this study design did not include an evaluation of women’s’ satisfaction relating to the co-designed strategies within the evidence base areas of changes. The long-term sustainability of the strategies, and their impact on immigrant women’s’ contraceptive choices. Such questions will be explored in future studies related to this project. Both LS and workshops were held online, which was something that we were hesitant to do at first. Yet, the online format worked much better than expected. The online format made it feasible to bring together midwives from various regions, which further enhanced the learnings. Not only across MCHs but also across regions in Sweden.

## Conclusion

This paper described the co-design process drawing on the Double Diamond model, with the aim to develop acceptable strategies to improve postpartum contraceptive services for immigrant women in Sweden as part of the IMPROVE-it project. The process allowed high participation and engagement and we managed to develop a series of practical approaches and strategies to improve the postpartum contraceptive counseling experience of both immigrant women and midwives. This shows that co-design as a method has the potential for further research and action in reproductive health. Our strategy was novel because we included both immigrant women and healthcare professionals on equal terms and because we engaged co-design participants from beginning to the end. Further research will assess the effectiveness of the developed interventions.

## Supplementary Information

Supplementary Material 1.

Supplementary Material 2.

## Data Availability

Data is available upon reasonable request from the corresponding author.
