# Supplementary material for: Co-designing postpartum contraceptive services with and for immigrant women in Sweden: lessons learned from the IMPROVE-it project
Source: BMC Health Serv Res. 2024 Oct 31;24:1325. doi: 10.1186/s12913-024-11709-2 (PMC11528989; doi:10.1186/s12913-024-11709-2)
Supplement: Supplementary file 1 — Supplementary Material 1. [file 12913_2024_11709_MOESM1_ESM.docx]

**Persona Somali speaking**

Amina is 37 years old and came from Somalia to Sweden 15 years ago. Amina met her husband Ismail in Sweden. It has been important for the couple to be able to support themselves and have their own home before having children. The couple uses condoms and interrupted intercourse as a method of contraception.

Amina has discussed and planned how many children she wants to have with her partner. She thinks it is important for the couple to share family issues as they have chosen to live their lives together. Amina also thinks it is important to exchange experiences, knowledge and information with her friends.

Amina says: “It's important for me not to have children too soon. For me and my family's health, I know that it is important to have at least two years between children.

Everything Amina knows about contraception she has learned from her Somali friends in Sweden. In Somalia she knew nothing about contraception. She has heard from her friends that contraceptives can cause cancer, infertility and side effects such as depression. She says she has several friends who have become pregnant despite using contraception.

Amina has been ill during her pregnancies and the pregnancies have been an anxious time for her and she is grateful for the care she has received. Questions about how many children Amina wants to have and when she plans to have the next child have contributed to a lot of stress and frustration during her pregnancies. She describes: -I worry about childbirth and they only talk to me about whether I want contraception and about a future child that doesn't even exist.

Amina often feels questioned about how many children she has. During her first delivery, which was a caesarean section, she was told that she will always have complicated deliveries and that she should keep her legs together for her husband. When Amina went home from the maternity ward, the staff said “See you again next year”.

It is important to Amina that her visits to the maternity ward focus on her health and that of her baby. During Amina's last pregnancy, the midwife provided information about what each visit would include and clearly explained why she was asking Amina different questions. They scheduled the contraceptive counseling for the postnatal check-up as Amina did not want to think about it during her pregnancy.

For the first time, Amina is thinking about using contraception as she is happy with the number of children she has and confident with the information she received from the midwife.

**Persona Arabic speaking**

Dana, a married woman with three children, had just fled Syria and settled in Sweden. She had her first two children immediately after marriage in Syria and recently delivered her third child in Sweden, with very short time between childbirths. She was relying on the natural method to control pregnancy, and she has realized that it was not a safe method.

 Now she is considering a more reliable way to control her pregnancies, she wants to more about how to increase the time between the pregnancies. Dana doesn’t know so much about contraception and has not received formal information about contraceptives, what she knows she has received long time ago in Syria from family and friends. Her knowledge is basic, she has heard about the methods but doesn’t know so much how they work.

One day, Dana was visited by two of her friends, Hala and Rania. Hala mentioned that she had started taking birth control pills but had noticed a significant gain in weight since then. Rania, on the other hand, warned Dana about the implant she had used to control pregnancy. Rania said that she had faced issues like hormones imbalance, irregular period and swollen abdomen, and had been told by her midwife that she needs to have it removed. Rania did also not know how to go about removing it, which was stressful for her.

Both Hala and Rania recommended that Dana use a copper IUD, but they also mentioned that many of their friends had experienced problems with it. Dana was feeling overwhelmed and confused. When the time came for her post-partum visit, Dana wanted to discuss more about her options with the midwife.

The midwife was kind and attentive, but Dana felt that she didn't fully explain the pros and cons of all the birth control methods, and she told her that Dana is the one who has to decide the method.  During the appointment, the midwife asked Dana about her private life with her husband and if there were any issues between them. Dana, feeling uncomfortable with the line of questioning, told the midwife that everything was fine and that the midwife didn't need to know about her private matters. Feeling like the midwife was missing the important topic of contraceptives and focusing more on her private life, Dana left the appointment without discussing in detail.

Feeling frustrated, Dana began to research birth control methods on her own, trying to understand the benefits and risks of each method. She knew that she needed to make a decision that was best for her and her family, but she didn't want to make a mistake. She started to talk with other women who also come from Syria who shared her experiences to understand their perspectives and experiences.
