# Supplementary material for: Co-designing postpartum contraceptive services with and for immigrant women in Sweden: lessons learned from the IMPROVE-it project
Source: BMC Health Serv Res. 2024 Oct 31;24:1325. doi: 10.1186/s12913-024-11709-2 (PMC11528989; doi:10.1186/s12913-024-11709-2)
Supplement: Supplementary file 2 — Supplementary Material 2. [file 12913_2024_11709_MOESM2_ESM.pdf]

IMROVE-it: Focus group discussion guide

1. What are your views on having children/having children?

-How do you/your partner talk about how many children you will have

- what is important?

2. How do you think about the number of children?

Most women in Sweden plan/decide how many children they want to have together - how do you think about it? How do you do it?

3. What do you know about contraception?

-what is good/bad about contraception

-what is good/bad about different contraceptive methods

-why good/bad to use contraception

4. Where did you get information about contraception?

- in your home country?

-in Sweden?

-between children?

- where did you get information

- experiences with digital information/advice

5. How do you feel about using contraception?

- Who decides?

-after the baby is born/emerged

-good/bad for women's bodies/health

If it does not come up

Here's what Somali men say

“Contraceptive methods are good to give women rest before the next pregnancy, but contraceptive methods are bad for women's bodies”

What do you think about it? In what way? How have you experienced it? Do you know anyone who has experienced it, in what way?

-Men are afraid that contraception will prevent women from having more children

What do you think about that?

In what way? How have you experienced it? Do you know someone who has experienced it, in what way?

6. What influences your decision to use contraception?

- What information is important about contraception

7. Who or what people do you listen to when you decide to use contraception?

-Partner/spouse, health professionals, family, friends, media, internet, other, etc.

8. a. How do you feel about your spouse/partner being involved when women talk about/ decide on contraception (for women) *.

b. How do you feel about being involved when your wife/partner talks about deciding on contraception? (for men)

9. How do you feel about talking about (deciding on) contraception when you are still pregnant?

-time/timing

-being involved in deciding what you want

-getting information

-make partner be involved

-good/bad for the female body/health

10. How do you feel about talking about contraception with health professionals like midwives and doctors?

- time for questions

-got to see pictures

-who decides

-plan to start using contraceptives

- at what time/timing

-during pregnancy-when the baby is in the womb

- What do you think about getting help with contraception and counseling via digital health services?

11. What do you think is important for midwives to know about Somali families when it comes to pregnancy and contraceptive use?

-What information should midwives give to Somali women who have recently had a baby

-What help do Somali women need?

- Somali men say this **

-time for questions

-be involved in deciding what you want

-get help with contraceptives that need to be inserted IUDs or rods

-book appointments

-help if contraception does not work

**-This is what Somali men say in a Swedish survey

“Men are not allowed to be present when midwives talk to women about contraception”

What do you say about that?

ex Men want to be involved in decisions about contraception

What do you say about that?

Follow-up questions:

Can you tell me more about?

Can you explain to me?

Can you give an example?

Mirror their words i.e. repeat the word they say themselves ....

Closing question:

Is there anything you would like to add that we have not discussed?

We are developing counseling for foreign-born women. Are there any of you who want to be involved and whom I can contact?

Telephone name
